# Tincture Antacrida

**Published:** 1901-08

**Authors:** N. F. Howard

**Affiliations:** Dahlonega, Ga.


### CORRESPONDENCE.

---

### TINCTURE ANTACRIDA.

*Editor Atlanta Journal-Record of Medicine:*

Some medical men have made inquiry of me in reference to the formula of the recipe of this tincture.

In the XIV. vol., page 642, of the *Southern Medical and Surgical Journal,* Augusta, Ga., of the year 1858, will be found an article from Prof. E. D. Fenner, in which he publishes the recipe of the tincture as follows:

℞ Gum guaiac .......... ℥ i.

Balsam canadens .......... ʒ i.

Ol. sassafras .......... ʒ ii.

Merc. corrosiv. sublimat .......... ℈ i.

Rect. spt. vini (alcohol) .......... ℥ viii.

"Dissolve the guaiac and balsam in one-half the spirit, and the corrosive sublimate in the other. Let the guaiac and balsam digest for several days; then pour off the clear liquor, mix with the sublimate and add the oil. Dose—Ten or twenty drops night and morning in a glass of wine or water, *pro re nata.*

Dr. Fenner estimates this medicine as invaluable in the treatment of dysmenorrhea and sterility in females. The doctor received it from his brother, who received it from their father, who read it in an English work as written by Dr. Falk. Will you please publish above for me. Would it not, however, be well to publish Dr. Fenner's entire article? It is very instructive and interesting, and would appear well in THE ATLANTA JOURNAL-RECORD OF MEDICINE.

N. F. HOWARD, M.D.,
Dahlonega, Ga.

